# Management of Crohn’s Disease in Adult Patients: A Contemporary Surgical Perspective

**DOI:** 10.3390/biomedicines14081774

**Published:** 2026-08-06

**Authors:** Constant Delabays, Emilie Zhu, Amaniel Kefleyesus, William Perry, James Ansell, Alain Schoepfer, Fabian Grass

**Affiliations:** 1 Department of Visceral Surgery, Lausanne University Hospital CHUV, University of Lausanne (UNIL), 1011 Lausanne, Switzerland; constant.delabays@chuv.ch (C.D.); emilie.zhu@ehc.vd.ch (E.Z.); amaniel.kefleyesus@chuv.ch (A.K.); 2 Division of Colon and Rectal Surgery, Mayo Clinic, Rochester, MN 55905, USA; perry.william@mayo.edu; 3 Department of Colorectal Surgery, University Hospital of Wales, Cardiff CF14 4XW, UK; james.ansell@wales.nhs.uk; 4 Department of Gastroenterology, Lausanne University Hospital CHUV, University of Lausanne (UNIL), 1011 Lausanne, Switzerland; alain.schoepfer@chuv.ch

**Keywords:** Crohn’s disease, surgery, Kono-S anastomosis, mesenteric excision, strictureplasy, biologics era

## Abstract

**Introduction**: Crohn’s disease (CD) remains associated with long-term morbidity despite biologic therapies. Surgery, historically considered a last-resort option, is increasingly integrated into disease management earlier. This review examines the evolving role of surgery in the biologic era. **Methods**: A focused narrative review of randomized controlled trials, meta-analyses, observational studies, and international guidelines addressing contemporary surgical strategies in CD was performed. **Results**: The LIR!C trial demonstrated that early ileocecal resection provides durable remission and improves long-term outcomes compared with biologic therapy in selected patients. The PISA II trial supported an early combined surgical and medical approach for perianal fistulizing disease. Preoperative optimization, including nutritional support and adjustment of immunosuppressive therapy, has become a cornerstone of perioperative management. Laparoscopic surgery remains the preferred approach whenever feasible, while robotic surgery is emerging as a promising platform that is expected to play an increasingly important role in the surgical management of CD. Postoperative recurrence remains a major challenge, prompting the development of innovative surgical strategies. Although the Kono-S anastomosis initially showed promising reductions in recurrence, recent prospective studies failed to confirm superiority over conventional techniques. Similarly, extended mesenteric excision did not demonstrate improved outcomes despite increasing evidence implicating the mesentery in CD pathogenesis. Strictureplasty remains an effective option for selected fibrotic small-bowel strictures. **Conclusions**: Surgery remains central to multidisciplinary CD management and offers the potential to modify disease when performed early in selected patients. The impact of innovative surgical strategies on postoperative recurrence remains uncertain, and ongoing trials are expected to further help with surgical decision-making.

## 1. Introduction

Crohn’s disease (CD) is a chronic inflammatory bowel disease that can affect the entire gastrointestinal tract and extraintestinal organs such as joints or skin. The typical onset of CD occurs in adolescence and young adulthood in Western countries. Incidence and prevalence have been increasing over the past decades, with a growing burden in newly industrialized countries undergoing rapid westernization [1,2,3]. CD is now recognized as a global disease, and this new epidemiology suggests industrialization and the Western lifestyle as triggers in CD pathophysiology.

CD is characterized by an intermittent, relapsing–remitting course that exerts a profound impact on patients’ quality of life and is associated with considerable long-term morbidity and thus a considerable healthcare burden. The management of CD has been radically transformed by the rise in biologic therapies, which have reshaped the natural history of the disease and reduced the need for surgery by nearly 30% [4]. With the emergence of biologic therapies, treatment pathways have become increasingly complex, requiring close multidisciplinary and interprofessional collaboration to optimize personalized patient care. In contrast, surgery has often been mistakenly perceived as a failure of treatment and a measure of last resort. Surgery was indeed traditionally reserved for complex cases such as intestinal fistulas and strictures, or for medically refractory disease courses. Despite these medical advances, surgery is still needed in about half of patients within 10 years of diagnosis, and up to 80% of CD patients will require intestinal resection during their lifetime [5,6,7,8].

In recent years, surgery has had to redefine its role, establishing its place within increasingly sophisticated decision-making algorithms to guide management. The role of upfront surgery, the technical aspects and configuration of the anastomosis, as well as the renewed focus on the role of the mesentery, are examples highlighting recent paradigm shifts in CD surgery. This review examines four critical questions: (1) What is the evolving role of surgery in the biologic era? (2) What is the place of surgical innovation and technical advances? (3) Do anastomotic configuration (Kono-S) and a mesenteric-inclusive approach impact postoperative outcomes? (4) How should surgeons interpret and integrate recent trial data into clinical practice?

## 2. Materials and Methods

We conducted a focused, expert-driven narrative review of the literature to evaluate the role of surgery in the management of CD in the current biologic era. Particular attention was given to postoperative recurrence, perioperative optimization, minimally invasive surgery, early surgical intervention, anastomotic techniques, mesenteric management, and bowel-preserving strategies such as strictureplasty. A targeted, non-systematic literature search was conducted between September 2025 and June 2026 using PubMed/Medline and SCOPUS. The search combined the terms CD, surgery, ileocolic resection, early surgery, laparoscopy, robotic surgery, postoperative recurrence, Rutgeerts’ score, Kono-S, strictureplasty, mesentery, and perioperative optimization. Relevant articles in English were considered and retrieved. In addition, the reference lists of relevant articles, meta-analyses, and international guidelines were manually screened to identify additional key publications through cross-referencing. This review was not designed as a systematic review. Therefore, the PRISMA methodology was not applied, and a flow diagram cannot be provided. Instead, studies were selected according to their methodological quality, scientific rigor, and relevance to contemporary surgical practice. Priority was given to landmark randomized controlled trials, high-quality prospective and retrospective cohort studies, and international guidelines that have shaped current evidence and clinical decision-making in CD surgery. Additional publications were included when they provided important historical context or addressed emerging concepts. The review intentionally focused on CD in the adult population. Studies exclusively involving pediatric patients (<18 years of age) were excluded since pediatric CD represents a distinct clinical entity with specific considerations regarding disease phenotype, manifestations according to growth patterns and development, surgical indications and implications, and long-term management.

The objective of this narrative review was to provide a comprehensive, clinically relevant, and evidence-based overview of current surgical management of CD.

## 3. Results

### 3.1. Place of Surgery: A Shift of Paradigm

The rise in biologic therapies has substantially improved disease control in CD while decreasing the occurrence of disease exacerbation. Although biologic treatments helped to decrease the short-term need for surgery, medical treatment still fails to achieve satisfying long-term disease remission in a relevant proportion of affected patients. Only one-third of CD patients receiving biologic therapies achieve the endpoint of mucosal remission [9]. Consequently, up to 80% of patients require surgical intervention during their lifetime, most commonly due to strictures, fistulas, or refractory disease [10,11]. The increased importance of surgical treatment has been recently demonstrated through two landmark studies: the LIR!C trial (Laparoscopic Ileocecal Resection versus Infliximab for terminal ileitis in Crohn’s disease) and the PISA trial (Perianal fistulas in Crohn’s disease, Seton versus Anti-Tumor Necrosis Factor α (Anti TNFα) versus Surgical closure following Anti-TNFα). The LIR!C trial randomly compared laparoscopic ileocolic resection vs. infliximab among patients with limited and non-stricturing ileocecal CD in whom conventional therapy had failed. This randomized controlled trial (RCT) revealed upfront surgery to provide comparable outcomes to infliximab in terms of quality of life and morbidity [12]. Interestingly, in the long-term follow-up study (median follow-up 63.5 months, interquartile range (IQR) 39–94), 22% of patients in the resection group required no additional treatment, 20% received only transient prophylactic immunomodulation, and none required additional surgery. On the other hand, almost half of patients in the infliximab group (48%) required resection, while the other half had to maintain, switch, or escalate anti-TNFα treatment [13]. More recently, the 10-year follow-up of the LIR!C trial confirmed the durability of these findings: therapy-free remission was achieved in 35.8% (95% Confidence Interval (CI) 2.6–50.1) of patients in the resection group compared to 13.2% (95% CI 6.1–23.2) in the infliximab group (difference 22.6%, 95% CI 7.8–36.8; *p* = 0.0038) [14]. Importantly, post hoc analyses suggested an age-dependent treatment effect, with younger patients deriving greater benefit from surgery in terms of sustained clinical remission (*p* interaction = 0.020). The estimated 10-year clinical remission rate for a 20-year-old patient was 50% (95% CI 34–69) with ileocecal resection versus 25% [13,14,15,16,17,18,19,20,21,22,23,24,25,26,27,28,29,30,31,32,33,34,35,36,37,38,39,40,41,42,43,44,45,46] with infliximab (difference 25%, 95% CI 2–48), compared with 37% (95% CI 25–52) with ileocecal resection versus 29% (95% CI 18–43) with infliximab for a 30-year-old patient (difference 8%, 95% CI −7 to 24). These results support early ileocecal resection as a potentially disease-modifying strategy, particularly in younger patients with limited ileal disease. These findings were corroborated by two meta-analyses, mainly on retrospective data [15,16]. Both publications demonstrated a lower rate of additional bowel resection, a longer period without resection or repeated resection, and a decreased need for further drug therapy. Of note, morbidity and quality of life were similar in both groups.

The second princeps trial evaluating early surgery for CD is the PISA trial. The PISA RCT focused on perianal CD and compared seton drainage to anti-TNFα therapy and a combination of surgical closure and short-course anti-TNFα therapy in patients with high Crohn’s fistulas, defined as fistulas located in the upper two-thirds of the external sphincter. The trial was terminated early due to futility: Seton drainage alone was associated with a high reintervention rate, and no conclusions were reached for the other two groups [17]. The PISA II trial hence compared anti-TNFα alone vs. surgical closure combined with short-term anti-TNF treatment [18]. The authors found the latter to induce a significantly higher magnetic resonance imaging (MRI)-proven long-term healing rate compared to anti-TNFα therapy alone (32% vs. 9% at 18 months; *p* = 0.005). Taken together, these findings emphasize the importance of an early combined medical and surgical approach in perianal CD.

This shift of paradigm, suggesting an increasingly important role of surgery at early disease stages, may lead to a higher rate of sustained remission. The underlying mechanisms are likely multifactorial and may involve a reduction in the inflammatory “disease burden” to reset the disease, thereby enabling more effective maintenance therapy at earlier disease stages. More specifically, resection of the mesenteric creeping fat, which secretes proinflammatory mediators, as well as the chronically inflamed bowel, which presents a defective mucosal barrier and increased intestinal permeability, may decrease exposure to luminal triggers that drive immunological response and intestinal inflammation [6,19]. The evolution in medical management from the traditional “step-up” approach to more proactive strategies such as “treat-to-target” [20,21] and “top-down” [22] currently used for medical treatment can also be applied to surgery. Early surgical intervention, as demonstrated in the LIR!C trial, represents a surgical equivalent of the top-down approach, offering comparable outcomes to biologic therapy while potentially avoiding prolonged exposure to costly and burdensome medications. In parallel, a “treat-to-target” strategy may also be incorporated into surgical care: systematic postoperative endoscopic monitoring and therapeutic escalation in the presence of early recurrence, as supported by the postoperative Crohn’s endoscopic recurrence (POCER) trial [23]. This RCT randomized 174 CD patients after intestinal resection to either risk-stratified treatment with 6-month colonoscopy and treatment intensification for endoscopic recurrence versus standard drug therapy alone. The endoscopy-guided strategy significantly decreased endoscopic recurrence compared to standard care (RR 0.73, 95% CI 0.56–0.96). Among high-risk patients (smokers, perforating disease, history of intestinal resection), adalimumab was superior to thiopurine in preventing endoscopic recurrence (13% vs. 39%, *p* = 0.02). These results established the principle that endoscopic monitoring at 6 months with treatment step-up based on findings may be more effective than medication alone in preventing postoperative recurrence. Both treat-to-target and top-down strategies are not mutually exclusive and can be integrated within the same treatment pathway as an opportunity for complementary, goal-directed management.

Early surgery may also be justified by the fact that delaying surgery in non-responders to medical therapy may lead to more advanced disease, requiring more extensive bowel resection and complex surgery, resulting in higher postoperative morbidity [24]. Moreover, unnecessary delays may elicit a substantial socioeconomic impact through an increased use of costly medical therapies.

### 3.2. Preoperative Optimization

At the time of surgery, CD patients present frequently with malnutrition, immunosuppressive therapy, anemia, physical deconditioning, and sarcopenia, all of which contribute to an increased risk of postoperative complications. Therefore, prehabilitation and optimization strategies play a pivotal role in improving surgical outcomes in this vulnerable population.

Nutritional management is a cornerstone of multidisciplinary care of patients with CD. Up to 85% of CD patients requiring surgery are affected by malnutrition. Several mechanisms contribute to malnutrition in patients with inflammatory bowel disease (IBD), including decreased nutritional intake, impaired nutrient absorption, chronic enteric protein losses, and elevated energy requirements resulting from the hypercatabolic state induced by inflammation [25,26]. A systematic review of 29 studies analyzing preoperative nutritional support in patients with CD found malnutrition to be associated with increased post-operative morbidity, while both enteral and parenteral routes were efficient in decreasing postoperative morbidity [27]. In a meta-analysis totaling 1111 patients, preoperative nutritional supplementation (enteral or total parenteral nutrition) significantly reduced overall postoperative complications compared to standard care (20.0% vs. 61.3%; OR = 0.26, 95% CI 0.07–0.99, *p* < 0.001) [28]. The European Crohn’s and Colitis Organization (ECCO) guidelines and the European Society for Clinical Nutrition and Metabolism (ESPEN) guidelines recommend systematic preoperative nutritional assessment in patients with CD awaiting surgery [8,29]. The preferred route is enteral nutrition, and total parenteral nutrition should be used when exclusive enteral nutrition is not tolerated. Nutritional optimization should ideally be undertaken within 2–6 weeks preceding surgery and should include correction of hypoalbuminemia, anemia, electrolyte disturbances, and vitamin deficiencies.

Active smoking is also associated with an increased risk of postoperative infectious complications (OR 1.3, 95% CI 1.18–1.54; *p* < 0.001), pulmonary complications (OR 1.87, 95% CI 1.39–2.51; *p* < 0.001), and hospital readmission (OR 1.58, 95% CI 1.15–2.16; *p* = 0.004) [30]. Furthermore, smoking represents a well-established risk factor for anastomotic recurrence and disease recurrence in the medium and long term. Therefore, smoking cessation at least 4 weeks before surgery is recommended to reduce postoperative complications [25,31].

For decades, corticosteroids represented the mainstay of medical therapy for CD. Although the therapeutic landscape has currently dramatically evolved with the introduction of biologics, corticosteroids continue to play an important role in the management of acute disease flares. Consequently, a substantial proportion of patients still receive corticosteroids in the preoperative period. However, prolonged corticosteroid exposure has consistently been associated with an increased risk of postoperative complications. In particular, treatment with ≥20 mg/day of prednisolone (or equivalent) for more than 6 weeks has been linked to an increased risk of postoperative septic complications [8]. A meta-analysis evaluating postoperative outcomes in patients with IBD undergoing abdominal surgery demonstrated that perioperative corticosteroid use was associated with a higher risk of overall postoperative complications (OR 1.41, 95% CI 1.07–1.87) and infectious complications (OR 1.68, 95% CI 1.24–2.28) [32]. This association appears to be dose-dependent, with high-dose corticosteroid therapy (>40 mg/day of prednisolone equivalent) conferring an even greater risk of postoperative complications (OR 2.04, 95% CI 1.28–3.26). Similar findings were reported in a large Cochrane review including 35 studies, which found a significantly increased risk of postoperative infectious complications among corticosteroid-treated patients (OR 1.34, 95% CI 1.25–1.44; *p* < 0.00001) [33]. Furthermore, a large American cohort study identified perioperative corticosteroid use as an independent risk factor for anastomotic leakage (OR 1.51, 95% CI 1.02–2.25) [34]. An interesting retrospective study revealed a dose-related effect on infectious complications: use of prednisone < 20 mg (OR 2.56, 95% CI 0.68–9.61), prednisone between 20 and 40 mg (OR 3.12, 95% CI 0.93–10.49), and >40 mg of prednisone (OR 9.16, 95% CI 1.51–55.42). There is a lack of data on cumulative dose and exposure. The American Society of Colon and Rectal Surgeons (ASCRS) guidelines advise to gradually reduce patients’ glucocorticoids at least to a daily dose <20 mg prednisolone before surgery to limit infectious risks [25]. This commonly used threshold of 20 mg/day of prednisolone is, however, based on observational evidence and expert consensus rather than randomized trials. Given the dose-dependent effect, supporting tapering to the lowest possible dose before surgery. Accordingly, the 2024 ECCO guidelines recommend reducing corticosteroid therapy whenever possible to minimize postoperative morbidity [8]. When steroid reduction is not achievable, a staged surgical approach with temporary fecal diversion should be considered, particularly in patients with additional risk factors for anastomotic leakage, such as smoking, malnutrition, anemia, or chronic bowel obstruction.

The introduction of immunomodulators represents a major advance in the treatment of CD by enabling long-term maintenance of remission while reducing corticosteroid dependence. Thiopurines were introduced in the late 1960s, followed by methotrexate in the 1990s. Current evidence suggests that perioperative use of purine analogs, including azathioprine and mercaptopurine, does not adversely affect postoperative outcomes. Indeed, a retrospective cohort study of 159 patients with IBD undergoing surgery has not found an increased risk of infectious complications among patients receiving thiopurines (adjusted OR 1.68, 95% CI 0.65–4.27) [35]. These findings were confirmed by a meta-analysis of 11 retrospective studies, which revealed no significant increase in the risk of overall postoperative or infectious complications among patients receiving thiopurines or cyclosporine [36]. Interestingly, although azathioprine and 6-mercaptopurine have short plasma half-lives, their immunosuppressive effects persist for several weeks due to intracellular accumulation of active metabolites. Consequently, temporary discontinuation shortly before surgery is unlikely to significantly impact the postoperative course. These different elements support the current ECCO and ASCRS recommendation to continue throughout the perioperative period [25,37]. A recent joint update of the WHO Surgical Site Infection (SSI) guidelines led by different European Societies confirms this strategy [38].

Although methotrexate is now used less frequently in the era of biologic therapies, it remains part of the therapeutic armamentarium for selected patients with CD. Evidence is therefore scarce. A retrospective study of patients with IBD undergoing abdominal surgery found no increased risk of either overall postoperative (OR 0.75, 95% CI 0.25–2.29) or infectious complications (OR 0.58, 95% CI 0.09–3.73) associated with perioperative methotrexate use [39]. These findings were subsequently confirmed by a meta-analysis conducted by the same group, which likewise demonstrated no significant increase in postoperative complications among patients treated with methotrexate for either IBD or rheumatic arthritis (OR 0.62, 95% CI 0.34–1.15). These different elements support the current ECCO and ASCRS recommendation to continue Thiopurine and Methotrexate throughout the perioperative period [25,37].

Since the introduction of anti-TNFα agents in the late 1990s, biologic therapies have transformed the management of CD. However, their perioperative safety remains controversial. While effective control of inflammation may reduce postoperative complications, its immunosuppressive effects have raised concerns regarding an increased risk of infectious complications and impaired anastomotic healing, leading to conflicting results across studies. Several meta-analyses and retrospective studies have reported inconsistent results [40,41,42,43]. These discrepancies likely reflect differences in population, outcome measure, and an inconsistent definition of outcome. The highest-quality evidence comes from a prospective multicenter observational study evaluating the impact of preoperative TNF inhibitor exposure on postoperative infectious complications in patients with CD [44]. Preoperative exposure, defined as treatment within 12 weeks preceding surgery, was not associated with overall infections (18.1% vs. 20.2%, *p* = 0.469; adjusted OR 1.05, 95% CI 0.72–1.54) or SSI (12.0% vs. 12.6%, *p* = 0.889; adjusted OR 1.25, 95% CI 0.79–1.96). Interestingly, detectable anti-TNF drug concentration was not associated with infectious complications or SSI in both univariate and multivariate analysis. For those reasons, the ECCO guidelines recommend against cessation of anti-TNF prior to surgery [8]. Ustekinumab is a monoclonal antibody targeting interleukin 12 and 23. A large Spanish multicenter retrospective study found that preoperative ustekinumab exposure within 12 weeks of surgery was not associated with an increased risk of 30-day postoperative complications [45]. A meta-analysis comparing ustekinumab to anti-TNF demonstrated no differences in complication rate (7.2%, 95% CI 3.0–16.4 vs. 11.9%, 95% CI 5.9–22.5; *p* = 0.4) [46]. Furthermore, a recent meta-analysis demonstrated that ustekinumab had the most favorable safety profile when compared to infliximab and vedolizumab in patients undergoing intestinal surgery for IBD [47]. Indeed, ustekinumab was associated with similar rates of overall postoperative complications (RR 0.55, 95% CI 0.20–1.57; *p* = 0.26) but a significantly lower risk of surgical site infections (SSI) (RR 0.35, 95% CI 0.17–0.73; *p* = 0.005). Although the evidence is less strong than with anti-TNF, current data suggest that cessation before surgery may not be necessary [8]. Finally, vedolizumab is a gut-selective anti-integrin monoclonal antibody. While early retrospective studies suggested an increased risk of postoperative infections with preoperative vedolizumab exposure, subsequent studies have consistently failed to confirm this association. Most recent meta-analyses similarly found no increased risk of postoperative morbidity, including overall complications (OR 1.04, 95% CI 0.48–2.24), infectious complications (OR 1.00, 95% CI 0.37–2.69), and surgical site infections (OR 1.45, 95% CI 0.33–6.32) [48]. Overall, decisions regarding the continuation or discontinuation of medical therapy in the perioperative period should not follow a one-size-fits-all approach. Decisions should be individualized, discussed within a multidisciplinary team, and guided by shared decision-making.

### 3.3. Surgical Approach and the Emergence of the Robotic Platform

Surgical approaches have considerably evolved over recent decades. The first revolution occurred in the 1990s with the widespread adoption of laparoscopic surgery, demonstrating significant advantages over open surgery in patients with CD, yielding shorter hospital stays, lower complication rates, and improved cost-effectiveness, while maintaining comparable long-term recurrence-free survival [49,50,51,52]. In the past two decades, robotic surgery has emerged as a major technical development in modern digestive surgery. Robotic-assisted surgery appears to offer significant advantages in colorectal surgery, including lower conversion and complication rates, as well as faster postoperative recovery, while achieving oncological outcomes that are at least equivalent to conventional approaches [38,53,54]. These benefits, however, may come at the cost of higher procedural expenses and longer operative times. This chapter provides an overview of the current evidence on robotic-assisted surgery for CD.

In a propensity score-matched analysis of a database including nearly 1400 patients undergoing ileocecal resection for CD comparing open, laparoscopic, and robotic approaches using the Hugo™ RAS system, robotic surgery was associated with a significantly lower conversion rate than laparoscopy (1.6% vs. 15.2%; *p* = 0.001), corresponding to a 89% reduction in the odds of conversion (adjusted OR 0.11; 95% CI 0.02–0.77; *p* = 0.027) [55]. Compared with laparoscopy, the robotic approach was also associated with a shorter length of hospital stay (median 6 days; IQR 5.0–7.0 versus 7 days; IQR 6.0–8.0; *p* = 0.049), a lower 30-day readmission rate (absolute risk reduction 16.1%; *p* = 0.025), and a lower rate of major complications (Clavien–Dindo grade ≥ III; *p* = 0.037), without increasing operative time. However, this study was limited beyond its retrospective design by the relatively small number of robotic cases (n = 62). In particular, the study population was heterogeneous, with a higher proportion of penetrating/fistulizing CD in the open surgery group, a phenotype known to be technically more challenging. These findings are supported by a recent meta-analysis including 3776 patients with CD, which demonstrated that robotic surgery was associated with fewer overall complications compared to laparoscopy (OR 0.56; 95% CI 0.31–0.99; *p* = 0.047) [56]. Operative time was, however, significantly longer (mean difference 51.8 min; 95% CI 32.0–71.6; *p* < 0.001). Similarly, another comprehensive meta-analysis including 13,225 patients with IBD reported lower conversion rates (OR −0.73; 95% CI −1.4–0.01; *p* = 0.02) and hospital stay (−0.65 days; 95% CI −1.25–0.05; *p* = 0.03) with robotic surgery, with comparable overall complication rates. However, again, robotic procedures were associated with significantly longer operative times (+67.3 min for ileocecal resection in Crohn’s disease; 95% CI 44.1–90.5; *p* < 0.01) [57]. Both meta-analyses were mainly based on retrospective studies, revealing a lack of high-quality evidence. A recent study of a national surgical outcome registry in the United States compared postoperative complications after total colectomy with end ileostomy for IBD (CD and Ulcerative Colitis) between open, laparoscopic, and robotic approaches [58]. Apart from a longer operative time, robotic surgery was associated with a higher rate of organ-space infection compared with laparoscopy (14.5% vs. 4.3%, *p* = 0.001). Despite a shorter length of stay, readmission rates were higher in the robotic compared to the laparoscopic group (20.5% vs. 9.9%, *p* = 0.01).

A recently published study including 314 patients further demonstrated similar recurrence rates following robotic and laparoscopic ileocecal resection after a median follow-up of 37.6 months [59], with endoscopic recurrence-free survival at 1 year of 71.8% vs. 73.0%, *p* = 0.82, and 3 years of 51.4% vs. 55.9%, *p* = 0.82. Likewise, no significant differences were observed in clinical (20.7% vs. 27.2%; *p* = 0.30) or surgical recurrence rates (3.7% vs. 0.9%; *p* = 0.11).

The 2020 ASCRS Clinical Practice Guidelines for the Surgical Management of CD endorse minimally invasive approaches to CD (GRADE 1b, strong recommendation, moderate quality evidence) [25]. Similarly, the 2024 ECCO Guidelines recommend laparoscopy as the preferred first-line approach for abdominal surgery in Crohn’s disease (evidence level 2) [8]. Neither guideline currently provides specific recommendations regarding the robotic platform. Therefore, the choice of surgical approach should be individualized according to patient characteristics, disease complexity, surgeon expertise and preference, and local resource availability. While robotic surgery appears to be a safe and effective alternative to laparoscopy, high-quality evidence remains limited, and formal cost-effectiveness analyses are needed to justify the increased financial burden related to acquisition and maintenance.

Beyond the mere choice of the surgical platform and the focus on surgery-related outcomes, it is important to increasingly consider patient-reported outcome measures (PROMs), especially in the complex setting of CD management, with a substantial impact of treatment decisions on daily life activities and quality of life of patients with CD. A recent systematic review revealed significant variations in how PROMs were used to evaluate perioperative CD outcomes [60]. Routine assessments using an internationally accepted online platform were suggested to monitor patients and support areas of treatment pathways that require further support to ensure high standards of care. Taken together, patient experience measures and a thorough evaluation of the psychological impact of treatment decisions need to be further studied and become an integral part of shared decision-making. Ideally, PROMs become an integral part of the design of future studies focusing on surgical management strategies.

### 3.4. Anastomotic Recurrence: A Surgical and Medical Challenge

Surgery in CD is not curative, and post-operative recurrence remains a major challenge for surgeons and gastroenterologists alike. The princeps study by Rutgeert et al. points out two important elements: (1) mucosal recurrence precedes symptomatic disease, and (2) the degree of inflammation is directly correlated to the risk of recurrence [61]. Interestingly, recurrence appears unrelated to residual disease or incomplete resection but reflects new inflammation instead [62]. We generally distinguish three sequential forms of postoperative recurrence in CD: endoscopic, clinical, and surgical recurrence. Endoscopic recurrence is assessed using the Rutgeerts’ score, which was originally developed to predict the risk of clinical recurrence following ileocolonic resection based on early postoperative endoscopic findings [62]. The modified Rutgeerts’ score (mRS) was later developed to differentiate i2 lesions into i2a and i2b in order to refine the classification (Table 1). Lesions classified as i2a are confined to the anastomosis and might reflect postoperative ischemic changes rather than recurrent inflammation. This distinction is important, since i2b lesions are associated with more severe disease and a higher risk of progression. Bachour et al. showed that mRS i2b lesions were independently associated with endoscopic disease progression, whereas i2a lesions were not predictive of severe recurrence [63]. This was also corroborated by a large retrospective Dutch study assessing the prognostic value of the mRS for long-term outcomes after primary ileocecal resection in patients with CD [64]. Predictive values of clinical and surgical recurrences revealed by this study are depicted in Table 2. Importantly, most clinical trials and guidelines generally define endoscopic recurrence as mRS ≥ i2, given that the prognostic significance of i2a remains debated. Clinical recurrence refers to the reappearance of symptoms attributable to CD, while surgical recurrence is defined as the need to repeat intestinal resection or strictureplasty due to recurrent disease. Without treatment within 1 year of resection, endoscopic recurrence is observed in 70 to 90% of patients, while clinical recurrence occurs in 20% to 40% [61,65]. Thereafter, the cumulative risk of clinically significant recurrent disease increases by approximately 10% with each subsequent year [66]. Consequently, most patients will ultimately experience recurrence, and reoperation rates of 50% to 70% are reported at 10 years after the first surgery [11,23].

The introduction of biologic therapies, particularly anti-TNFα agents, has reduced postoperative recurrence rates [23,67]. However, endoscopic recurrence still ranges from 30 to 70% of patients at 1 year of bowel resection [68]. Early endoscopic surveillance is recommended by the ECCO guideline within 6–12 months after surgical resection in order to identify patients presenting with disease recurrence, enabling timely, prompt, and active intervention [7,8]. Several risk factors for recurrence have been identified and may help stratifying this risk, including smoking (clinical recurrence; OR 2.15; 95% CI 1.42–3.27; *p* < 0.001) [69], penetrating disease (surgical recurrence; OR 1.41; 95% CI 1.02–1.94, *p* = 0.04) [70], or history of previous resection (surgical recurrence; OR 1.7; 95% CI 1.23–2.38; *p* = 0.002) [71].

The anastomosis plays a central role in postoperative disease recurrence, with up to 90% of recurrences occurring at or adjacent to the anastomotic site [27]. Strategies to reduce recurrence include modification of risk factors, medical prophylaxis, and optimized surgical technique. The type of anastomosis has been extensively debated. Historically, hand-sewn end-to-end (HSEEA) and stapled side-to-side anastomosis (SSSA) have been compared. The Crohn Anastomosis Study Trial (CAST) did not reveal significant differences between the two techniques in terms of postoperative disease recurrence at 1 year [72]. However, there is concern about safety: one meta-analysis revealed HSEEA to be associated with an increased risk of anastomotic leak when compared to SSSA (OR 4.37; 95% CI 1.3–14.72; *p* = 0.02) [73]. These results were supported by a second meta-analysis confirming the superiority of SSSA in terms of overall complications, occurrence of anastomotic leak, disease recurrence, and need for reoperation due to recurrence [74]. For these reasons, ECCO guidelines recommend the SSSA technique for small bowel or ileo-colic resection for CD [7].

Beyond pure technical aspects, the anastomosis also reflects the surgeon’s experience, training background, and personal convictions, as illustrated by the wide heterogeneity of anastomotic techniques in clinical practice [75]. Anastomotic recurrence remains a major challenge in CD, motivating surgeons to imagine, develop, and refine novel techniques to address the inherently high recurrence rates at the anastomotic site. Main examples of this drive towards innovation in recent decades are the development of the Kono S anastomosis, as well as the strategy of extended mesenteric resection.

### 3.5. Kono-S Anastomosis: Evolving Evidence and Current Appraisal

**Figure 1:**
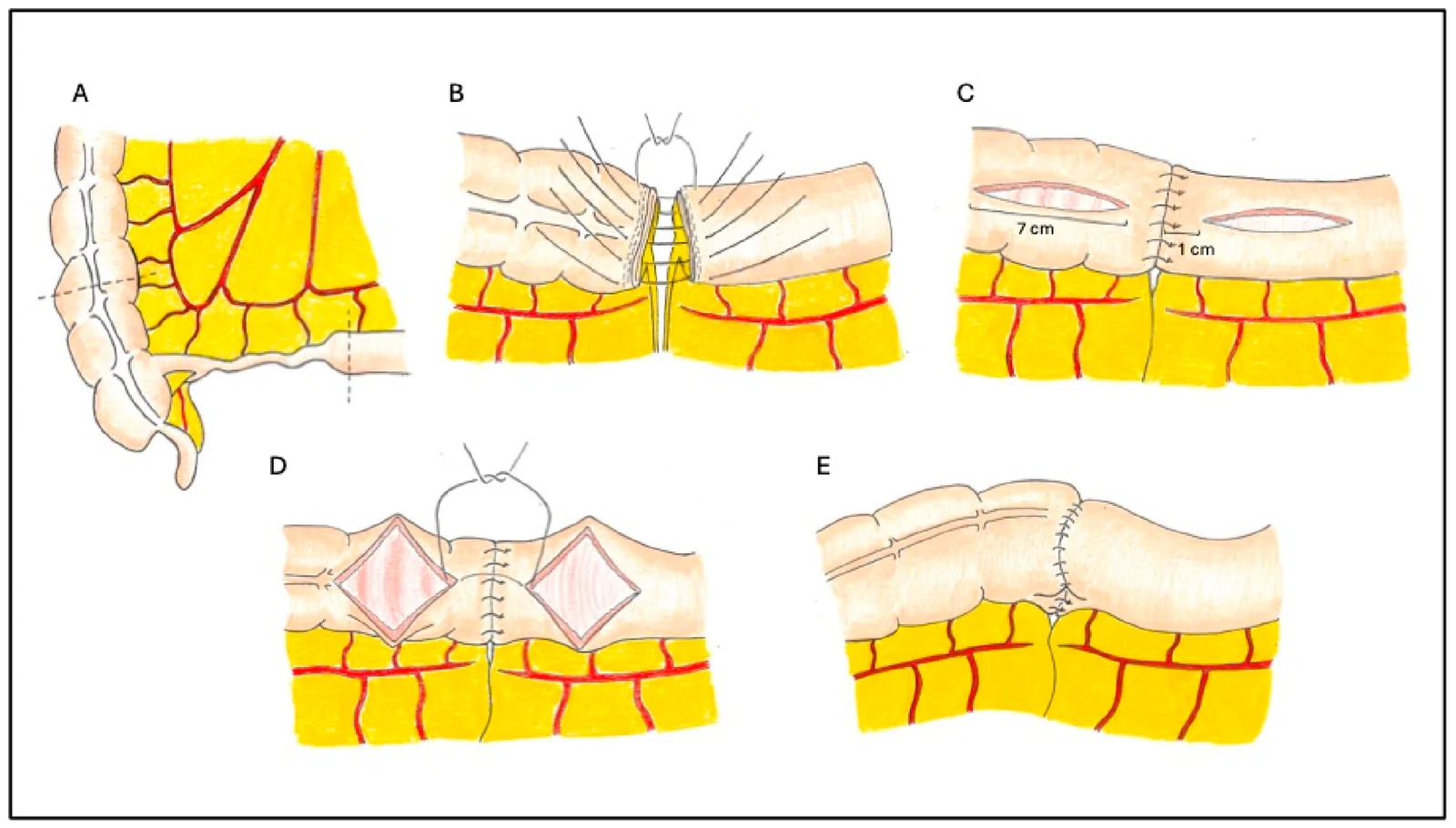
Kono-S anastomosis. (**A**) transection of small and large bowel in healthy tissue with removal of the diseased part, with the mesentery centered on the staple line, perpendicular at 90 degrees. (**B**) The two bowel segments are then approximated with sutures, forming a supporting column that provides mechanical and dimensional stability to the anastomosis. (**C**) A longitudinal enterotomy is then performed on each bowel segment, approximately 1 cm away from the supporting column, creating a transverse opening of 7 cm. (**D**) A hand-sewn, antimesenteric anastomosis is then fashioned. (**E**) Final configuration of the anastomosis.

The Kono-S anastomosis was first performed in Japan in 2003 by Professor Kono and his team, with the aim of reducing local recurrence [76]. The development of this anastomosis is based on several key principles: a large anastomosis, positioned away from the mesentery and supported by a three-dimensional structure anchored on a “supporting column” [76,77]. The main steps are illustrated in Figure 1. First, the small bowel is transected in healthy tissue with removal of the diseased part, with the mesentery centered on the staple line and perpendicular at 90 degrees. Vascularization and innervation are preserved as much as possible by dividing the mesentery close to the bowel wall. The two bowel segments are then approximated with sutures, forming a supporting column that provides mechanical and dimensional stability to the anastomosis. A longitudinal enterotomy is then performed on each bowel segment, approximately 1 cm away from the supporting column, creating a transverse opening of 7–8 cm. A hand-sewn, antimesenteric anastomosis is then fashioned.

The supporting column plays a central role in this anastomosis. Its rigidity is thought to help maintain both the diameter and orientation of the bowel lumen, thereby preventing distortion and restenosis even in the event of local recurrence. Recurrence typically develops on the mesenteric side of the anastomosis, highlighting the role of the mesentery in the pathogenesis of CD. By excluding the mesenteric side through the column, this technique intends to reduce exposure to inflammation and thus the risk of recurrence. Moreover, the Kono-S preserves both adequate blood supply and innervation of the anastomosis, two crucial elements for the healing process that are often impaired in CD and associated with anastomotic recurrence [78]. The final shape of the Kono S anastomosis is similar to an end-to-end anastomosis, with a wide lumen, which is easily accessible to endoscopy.

The first data about this new anastomosis were published in 2011 in a retrospective comparative study evaluating Kono-S versus conventional anastomoses [76]. Although endoscopic recurrence rates at 1 and 5 years were similar, surgical recurrence, defined as the need for reoperation with resection of the anastomosis, was absent in the Kono-S group compared to 15% in the conventional group (*p* = 0.0007). Furthermore, the severity of endoscopic lesions was lower in the Kono-S group (median Rutgeerts’ score at 5 years: 2.6 [range 1–4] vs. 3.4 [range 2–4], *p* = 0.008). While this technique could not entirely prevent endoscopic recurrence, it was suggested that it may help to prevent progression to surgical recurrence, regardless of postoperative medical therapy. The study, however, was limited by its small sample size and the monocentric retrospective design. Moreover, more patients in the Kono S group received postoperative infliximab therapy (42% vs. 16%, *p* = 0.0025), while the type of anastomosis was not standardized in the conventional group (end-to-end, functional end-to-end, side-to-side).

This initial publication generated enthusiasm within the surgical community and inspired several follow-up studies. The Surgical Prevention of Anastomotic Recurrence by Excluding Mesentery in Crohn’s Disease (SuPREMe-CD) trial was the first RCT on the subject [79]. In this Italian monocentric RCT, Kono-S anastomosis was compared with conventional side-to-side stapled anastomosis in 79 patients undergoing ileocecal resection for CD. Patients with Kono-S anastomosis had a significantly lower risk of endoscopic recurrence in the per protocol analysis at 6 months (22.2% vs. 62.8%; OR 5.9, 95% CI 2.17–16.05; *p* < 0.001) and at 18 months postoperatively (25% vs. 67.4%; OR 6.21, 95% CI 2.31–16.69; *p* < 0.001). Logistic regression confirmed anastomotic technique as the sole predictor of endoscopic recurrence, and recurrence-free survival was higher in the Kono-S arm (HR 0.36, 95% CI 0.14–0.94; *p* = 0.037). No significant differences were observed in postoperative morbidity or safety. However, this trial was limited by its single-center design, short follow-up (recurrences typically occur 5–10 years after surgery), lack of standardized postoperative medical therapy, and reliance on an exclusively endoscopic primary endpoint. A secondary study involving the same patient cohort found that patients with Kono-S reported improved bowel symptoms and social functioning compared to those receiving side-to-side anastomosis [80].

The first international meta-analysis of Kono-S anastomosis, including 9 studies and 676 patients, reported highly encouraging results [81]: pooled analysis showed 0% surgical recurrence, 5% endoscopic recurrence after sensitivity analysis, and low morbidity. However, these findings were limited by the retrospective, non-comparative, and heterogeneous nature of most included studies, as well as variability in perioperative medical management. Another meta-analysis specifically focused on endoscopic recurrence rates according to anastomotic type [82]. The authors demonstrated a trend favoring Kono-S, with a pooled endoscopic recurrence rate of 24.7% (95% CI, 6.8–49.4%) in the Kono-S group versus 42.6% (95% CI, 32.2–53.4%) in conventional anastomosis groups. Again, this meta-analysis was mainly based on observational studies and included only two comparative studies with control groups. More recently, a larger meta-analysis including 15 comparative studies (1501 patients, of whom 765 underwent Kono-S) demonstrated significantly lower rates of endoscopic recurrence (41% vs. 48%; RR 0.86, 95% CI 0.73–1.00; *p* = 0.05), surgical recurrence (2.7% vs. 21%; RR 0.13, 95% CI 0.06–0.30; *p* < 0.001), and anastomotic leak (1.7% vs. 4.9%; RR 0.37, 95% CI 0.19–0.74; *p* = 0.005) compared to conventional techniques [83]. Nonetheless, the overall quality of evidence remains limited due to the predominance of retrospective studies, reliance on historical controls, inclusion of only two RCTs, and lack of long-term follow-up.

These promising initial results have been tempered by more recent studies. In a monocentric comparative study of 85 patients, a French group failed to demonstrate the superiority of Kono-S [84]. Endoscopic recurrence rates at 6 and 12 months, as well as clinical recurrence rates, were similar between study groups. Multivariable analysis revealed anti-TNFα therapy as an independent protective factor, whereas no impact of the anastomotic technique on recurrence was found. These findings were corroborated by the KoCoRICCO study, a larger multicenter prospective study [85]: after matching with a historical cohort, no significant differences in endoscopic recurrence were observed at the first surveillance colonoscopy (47.5% in the Kono-S group vs. 44.3% in the control group). The discordance between early Japanese and recent Western trials may reflect several factors. First, Kono-S requires specific technical expertise, with superior outcomes reported in high-volume centers. Second, high rates of postoperative anti-TNFα therapy in recent trials (>90% in the GETAID study) may underestimate technique-related benefits. Third, Kono’s original data showed divergence in surgical recurrence only after 5 years, whereas current RCTs report on shorter follow-up with endoscopic endpoints at 6–18 months.

Given these conflicting results, there is a clear need for high-quality evidence. A large, multicenter, international RCT was initiated in 2014 with the aim of comparing postoperative CD recurrence between the Kono-S procedure and side-to-side functional end anastomosis, and to evaluate surgical recurrence rates at 60 and 120 months (NCT 03256240). Preliminary results, presented at the 2024 ECCO congress, showed no difference in endoscopic recurrence between the two groups at 3–6 months [86], as well as at 12–18 months [87]. Several centers are still recruiting, and results are expected by the end of 2026. A second large-scale RCT is underway, with two parallel arms designed to determine which anastomotic technique (hand-sewn (end-to-end vs. Kono-S) or stapled side-to-side) is superior in terms of endoscopic recurrence, gastrointestinal function, and healthcare costs [88]. The Mesenteric Excision and Kono-S anastomosis Trial (MEErKAT) is a multicentric RCT from the United Kingdom randomizing patients with CD undergoing ileocecal resection into four groups: (1) Kono S + close mesenteric resection; (2) Kono S + extended mesenteric resection; (3) standard anastomosis + extended mesenteric resection; and (4) standard anastomosis + close mesenteric resection [89]. Recruitment is expected to be completed in April 2026.

### 3.6. The Mesentery: Marker or Driver of Inflammation?

Mesenteric involvement in CD was first described by Burrill Crohn in 1932, and creeping fat is now recognized as a hallmark of macroscopic disease. In the early era of surgical CD management, surgeons performed radical mesenteric resections, which were associated with substantial morbidity and mortality [90]. For this reason, interest in the mesentery declined within the medical community, which subsequently considered the mesentery a passive and inert anatomical support structure. Nowadays, current ECCO guidelines recommend preserving the mesentery during surgery to minimize potential injury to the neurovascular supply and to preserve bowel perfusion and length. However, recent evidence has emerged suggesting that the mesentery may actively participate in CD pathogenesis and promote postoperative recurrence [91]. This evolving understanding has motivated re-exploration of mesentery-inclusive resections with modern surgical expertise. This section summarizes the key studies, underlying mechanisms, and ongoing debates on the topic.

The mesentery is now recognized as an immunologically active and functional structure, with its adipose tissue driving immune responses and producing an array of pro- and anti-inflammatory mediators [6,92,93]. Mesenteric thickening and mesenteric fat wrapping, defined as the extension of mesenteric fat onto the intestinal surface, are considered pathognomonic features of CD [93], correlating with disease severity and extension [92]. Several studies investigate the impact of an altered mesenteric and lymphatic system on transmural inflammation [94,95]. Mesenteric thickening may result from mispatterned and ruptured lymphatic vessels, leading to the activation of inflammatory pathways [96]. Moreover, an increased density of mesenteric lymphatic vessels has been reported in CD, where it is associated with creeping fat and intestinal granulomas [97]. Taken together, these findings support the role of the mesentery in CD physiopathology as a source of pro-inflammatory activity, therefore contributing to disease recurrence. The hypothesis that complete mesenteric excision could reduce postoperative recurrence of CD after intestinal resection has increasingly emerged within the scientific community.

In 2018, Coffey et al. published a landmark cohort study evaluating the impact of mesenteric resection on postoperative CD recurrence [98]. The study compared an ileocolic resection strategy that included the adjacent mesentery (mesenteric-inclusive resection; n = 34) to conventional mesentery-sparing resection (n = 30). Surgical recurrence was significantly lower in the mesenteric-inclusive resection group (2.9% vs. 40%, *p* = 0.003). The authors developed a mesenteric disease activity index, using fat wrapping and mesenteric thickening as severity parameters, which correlated with the CD Activity Index (CDAI). They also measured circulating fibrocyte levels in the mesenteric resection group. The circulating fibrocyte percentage was increased in CD patients compared to healthy patients and decreased after intestinal and mesenteric resection. More specifically, CD45 + αSMA + fibrocytes were identified exclusively in CD patients, within and near mesenteric vessels, in clusters at the intestinal surface, and in the connective tissue septa of the outer muscle layers in adjacent intestine. These findings suggest that the mesentery may serve as a reservoir for disease-promoting cells and cytokines, contributing to the ongoing interest regarding the role of the mesentery in postoperative recurrence. However, these results should be interpreted with caution due to several limiting factors: the absence of randomization, the small sample sizes in both groups, the comparison with a historical cohort with different post-operative surveillance and limited prophylactic medical options, and the longer follow-up. Moreover, the mesenteric resection group received more biologic therapy before surgery and exhibited a different disease phenotype, with fewer patients presenting stricturing or penetrating disease patterns, both representing risk factors for recurrence.

More recently, the SPICY trial (Mesenteric SParIng Versus Central mesenterectomY), an international randomized controlled trial, was conducted at six tertiary centers in the Netherlands and Italy in order to evaluate whether extended mesenteric resection improves outcomes compared with conventional mesentery-sparing resection in patients undergoing primary ileocolic resection for CD [99]. The main outcome was endoscopic recurrence at 6 months, defined by a modified Rutgeerts’ score of at least i2b. The trial failed to demonstrate superiority of extended mesenteric excision (42% in the extended mesenteric resection group vs. 43% in the mesentery sparing resection group, *p* = 1.0). These results were supported by a recent meta-analysis by Mostafa et al., who demonstrated comparable rates of endoscopic and surgical recurrence between the two approaches [100]. The SPICY trial results, despite the compelling rationale, warrant careful interpretation. First, the trial was powered to detect a large absolute reduction in endoscopic recurrence at 6 months (25-percentage-point absolute reduction; 60% vs. 35%). While the target sample size was reached, the observed recurrence rate (43%) was substantially lower than anticipated, likely reducing the study’s statistical power. Consequently, smaller, yet clinically meaningful, differences may have remained undetected. Moreover, the trial compared anatomically defined resection strategies based on predetermined vascular ligation points. This approach may not capture individual variations in mesenteric disease burden. Additionally, the 6-month endoscopic endpoint might not reflect sustained clinical and surgical longer-term outcomes. Furthermore, patients presenting with a considerable amount of creeping fat may represent a distinct subset benefiting from mesenteric-inclusive surgery. This potential benefit could hence be diluted in unselected populations. Future trials incorporating mesenteric phenotyping through preoperative MRI or histological analysis should focus on more specific and homogeneous patient populations, more likely to benefit from targeted mesenteric resection strategies. Finally, the SPICY trial also raises questions about the safety of extended mesenteric resection related to extensive bowel devascularization as a result of resection. Indeed, the authors reported a non-significantly increased rate of anastomotic leakage in the intervention group (8%, n = 5 vs. 2%, n = 1; RR 4.92; 95% CI 0.59–41.01, *p* = 0.2). The numerical imbalance raises concerns, and the absence of a statistically significant difference should be interpreted with caution, as the study was likely underpowered to detect differences in postoperative complications.

Several RCTs are underway to analyze the impact of mesentery-inclusive resection strategies, including the aforementioned MEErKAT trial assessing the role of both Kono S anastomosis and the extent of mesenteric resection [89]. Another trial in the United States is currently investigating endoscopic CD recurrence after ileocolic resection, comparing high vascular ligation to a mesentery-sparing approach (SPARES trial, NCT04578392). These trials will help refine surgical resection strategies and further clarify whether the mesentery acts primarily as a marker of inflammation or as an active driver in the pathogenesis of disease flares. Until such evidence, conservative resection of mesentery with close to the bowel resection remains the standard of care, as recommended by the ECCO guidelines [8].

### 3.7. Strictureplasty and Resection Margins in the Biological Era: Current Evidence

Up to 30–50% of patients with CD will develop stricturing complications over time, many of which become clinically significant and require intervention [101,102]. Strictures in CD can be classified as inflammatory, fibrotic, or mixed. Differentiating between these subtypes may be challenging and requires a combination of the clinical course, imaging findings, and biochemical markers [103]. While systemic therapies are effective in inflammatory strictures, they are limited or ineffective in fibrotic strictures. Among available medical options, anti-TNFα agents appear to provide the most significant benefit. However, a substantial proportion of patients will ultimately require more invasive management, either endoscopic (e.g., balloon dilation or stricturotomy) or surgical [103].

Surgical management of CD-related strictures includes segmental resection and strictureplasty. Strictureplasty techniques were developed as bowel-sparing techniques, aiming to preserve intestinal length in patients with recurrent or extensive disease. This approach allows luminal widening without resection and can be repeated at multiple sites, thereby reducing the risk of short bowel syndrome associated with cumulative resections, and is typically indicated in patients with symptomatic, fibrotic, and segmental strictures. Strictureplasty should be avoided in cases of active inflammation, fistulizing disease, abscess, suspected malignancy, or extensive strictures (>60 cm) [104,105]. Moreover, it is not recommended for gastric, ileocolic, or colonic strictures [104]. Several techniques have been described, primarily depending on the structure length. The most commonly used are the Heineke-Mikulicz strictureplasty (<10 cm), Finney strictureplasty (10–25 cm), and non-conventional techniques such as the Michelassi strictureplasty, which can be applied to longer segments of up to 60 cm [103,106,107]. These techniques have been shown to be safe and effective, with both short- and long-term outcomes comparable to those of conventional resection [108,109]. Rates of reoperation are similar to those observed after resection [110], and functional outcomes and quality of life are also comparable [111]. Furthermore, a meta-analysis including 1612 patients found no significant difference between conventional and non-conventional strictureplasties in terms of early or late complications [109]. The 2024 ECCO guidelines recommend strictureplasty as an alternative treatment option to resection for small-bowel disease [8]. In more recent decades, however, strictureplasty techniques became less popular [112,113]. This change in surgeon preference may be explained by decreased awareness of the risk of short bowel syndrome, driven by the efficiency of emerging medical treatment options. Another explanation may relate to a population-level shift in CD phenotype following the introduction of biologic therapies, with a reduced incidence of stricturing disease. Indeed, data from the Veszprem Cohort, a large prospective Hungarian cohort, showed significant decrease in the probability of behavior progression from inflammatory (B1) to stricturing disease (B2) since the introduction of biologics: the 10-year risk declined from 44.3% in the pre-immunomodulator era to 30.6% in the pre-biologic era and further to 16.1% in the biologic era (*p* < 0.001) [114]. These results were confirmed in a French population-based study [115]. Interestingly, the impact on penetrating disease is less clear. Jones et al. demonstrated in a large national database that since the introduction of anti-TNFα, the rate of surgical procedures for Crohn’s disease-related fistula of the small intestines had significantly increased in frequency [116].

Most available data originate from cohorts treated prior to the widespread use of biologic therapies [117,118,119]. Consequently, there is a lack of contemporary evidence regarding the outcomes and current role of strictureplasty in the modern therapeutic era, as well as the impact of new treatments on the risk of recurrence after this specific type of surgery. The group from the Cleveland clinic conducted a retrospective review of 71 patients who underwent strictureplasty. Patients were categorized into two groups depending on whether they were treated with biologics at the time of surgery or not. Patients treated with biologics at the time of surgery had a slightly longer median time to recurrence (4.7 vs. 4.4 years; *p* = 0.004) [120]. Another retrospective study reporting on 266 patients who underwent a total of 718 strictureplasties investigated risk factors for CD recurrence. Non-conventional techniques, stricturoplasty performed on a previous anastomosis, and exposure to biologics after stricturoplasty were all associated with recurrence [121]. However, these findings should be interpreted with caution, given the small sample size, retrospective design, and lack of details regarding indications for biologic therapy, potentially related to more aggressive disease requiring more aggressive treatment. Interestingly, in this same cohort, smoking was associated with a higher risk of recurrence in milder disease.

Strictureplasty, in combination with modern biologic therapies, appears to be safe and effective. However, the precise impact of biologics on recurrence rates remains unclear and warrants further investigation through well-designed prospective studies. Strictureplasty should remain part of the surgical armamentarium for selected indications, particularly in jejunal strictures, whether short or extensive. These techniques should also be incorporated into surgical training programs.

Another surgically modifiable factor that may influence postoperative outcomes in CD is related to the resection margin. Historically, surgery for CD involved wide intestinal resections with considerable margins of healthy bowel in an attempt to reduce postoperative recurrence. This strategy was, however, associated with high morbidity and mortality, as well as an increased risk of short bowel syndrome [90]. Moreover, wide excision does not prevent recurrence. A landmark study published in 1996 conducted at the Cleveland Clinic demonstrated indeed that recurrence rates were not influenced by the extent of resection margins [122]. In this RCT, including 131 patients undergoing ileocecal resection, limited resection (2 cm from the macroscopically involved segment) was compared to extended resection (12 cm margins). The authors found no differences between the two groups (25% vs. 18%, *p* = 0.38). Recurrence of CD may therefore be unaffected by the extent of the margin from macroscopically involved bowel. Furthermore, recurrence rates also do not increase when microscopic CD is present at the resection margins. As a result, surgical strategies have progressively shifted towards limited, bowel-sparing resections, with extensive resection margins to be restricted to macroscopically involved segments only.

## 4. Discussion

**Figure 2:**
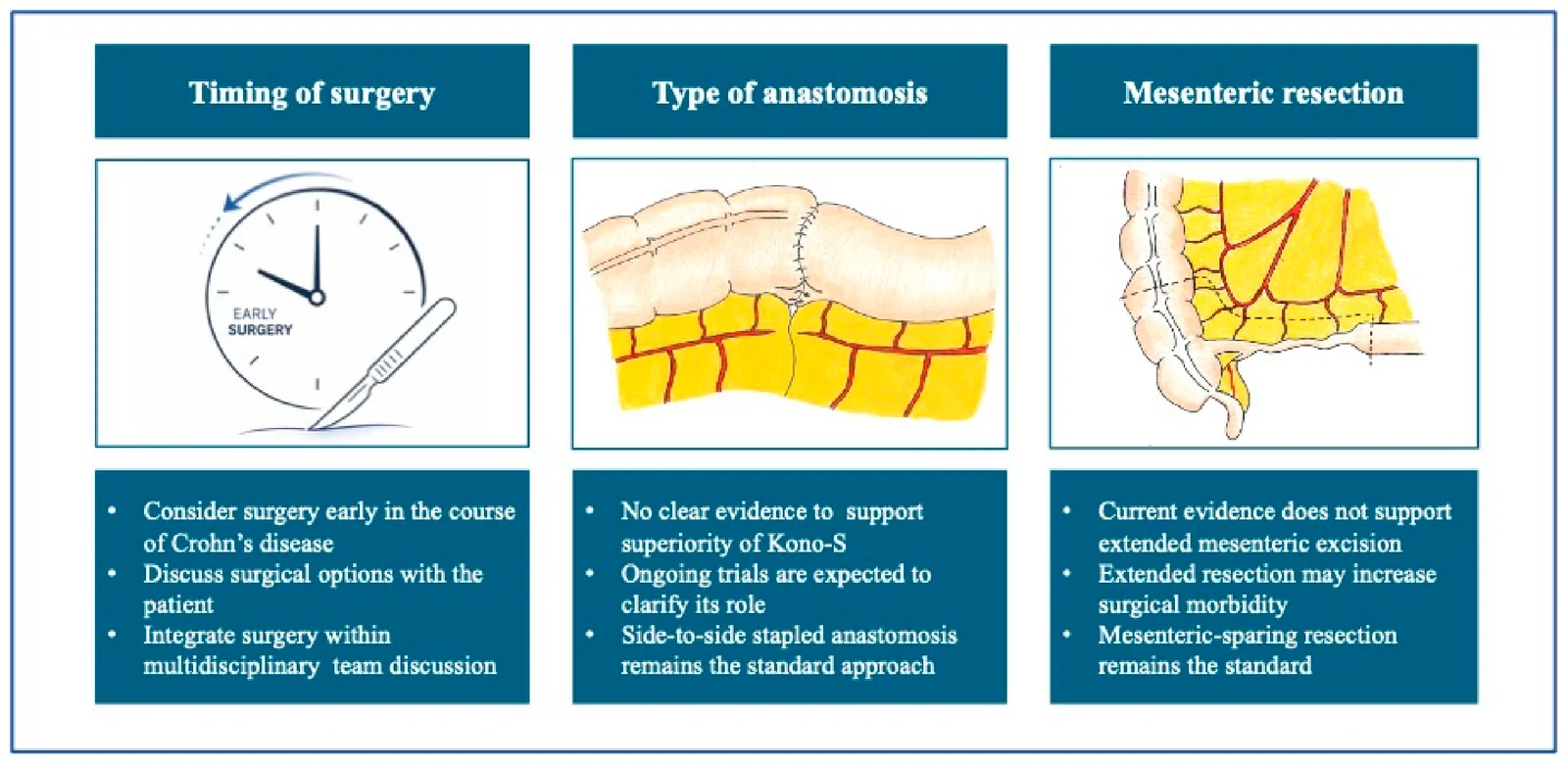
Summary of evidence of contemporary surgical perspective.

Despite the rise in biologic therapies, surgery still has a central role within increasingly complex, multidisciplinary, and personalized treatment pathways (Figure 2). Over the past decades, surgery has undergone a profound shift in paradigm, moving from the last resort and salvation options for advanced disease to a strategy of early and upfront surgery. The removal of the disease burden may “reset” treatment opportunities, with non-inflammatory tissue potentially being more responsive to subsequent medical therapy. Hence, it appears crucial to discuss surgical options early in the CD course, both with the patient and with a multidisciplinary team. Despite the introduction and development of innovative and promising biological treatments, postoperative recurrence at the anastomotic site remains a major challenge, motivating technical innovations targeting both anastomotic configuration and mesenteric management. Despite initial enthusiasm regarding the KONO S anastomosis within the surgical community, more recent high-quality studies have tempered these early, promising results and failed to demonstrate a clear advantage. The mesentery has recently been re-evaluated in CD pathogenesis, with growing evidence suggesting that mesenteric inflammation may sustain disease activity. Accordingly, mesenteric excision during intestinal resection has been proposed as a potential way to reduce postoperative recurrence. Once again, after initial promising results in favor of extended mesenteric resection, more recent studies did not demonstrate superiority of this approach compared to mesentery-sparing resections. Ongoing trials such as the MEErKAT and the international Kono-S study are expected to settle the debate in the next few years and clarify whether specific patient subgroups may particularly benefit from these technical innovations. Until such evidence emerges, judicious application of established principles—side-to-side stapled anastomosis, mesenteric preservation of vascular integrity, and systematic postoperative monitoring—remain cornerstones of surgical management.

## Figures and Tables

**Table 1:** Modified Rutgeert’s score.

i0		No lesion in the neoterminal ileum
i1		≤5 aphthous lesions in the neoterminal ileum
i2	>5 aphthous lesions with normal mucosa between the lesions, or skip area of large lesions, or lesions confined to the ileocolonic anastomosis	
	i2a	Lesions confined to anastomosis (including anastomotic stenosis)
	i2b	>5 aphthous ulcers or larger lesions, with normal mucosa in between, in the neoterminal ileum (with or without anastomotic lesions)
i3		Diffuse aphthous ileitis with diffusely inflamed mucosa
i4		Diffuse inflammation with large ulcers, nodules, and/or narrowing in the neoterminal ileum

**Table 2:** Risk of clinical and surgical recurrence according to the index mRS.

Index mRS	Clinical Recurrence	Surgical Recurrence	Progression to Severe Endoscopic Recurrence (mRS ≥ i3)
i0	42.2%	7.7%	21.1%
i1	53.7% aHR 1.7 (95% CI 1.2–2.4)	5.3%	33.9%
i2a	58.5% aHR 1.7 (95% CI 1.2–2.4)	12.9%, non-significant	26.8%
i2b	80.2% aHR 4.4 (95% CI 3.2–6.0)	19.1% aHR 3.0 (95% CI 1.5–5.6)	33.3%
i3	79.4% aHR 3.6 (95% CI 2.5–5.2)	28.8%; aHR 4.0 (95% CI 2.0–7.9)	-
i4	95.3% aHR 7.3 (95% CI 4.8–10.9)	47.8%; aHR 8.0 (95% CI 4.0–16.0)	-

Note: mRS: modified Rutgeerts’ score; aHR: adapted hazard ratio, CI: Confidence interval. Summary of the findings reported by Bak et al. [64].

## Data Availability

No new data were created or analyzed in this study. Data sharing is not applicable to this article.

## Abbreviations

The following abbreviations are used in this manuscript:

CD	Crohn’s Disease
RCT	Randomized Controlled Trial
MRI	Magnetic Resonance Imaging
IBD	Inflammatory Bowel Disease
ECCO	European Crohn’s and Colitis Organization
ESPEN	European Society for Clinical Nutrition and Metabolism
ASCRS	American Society of Colon and Rectal Surgeons
TNF	Tumor Necrosis Factor
PROM	Patient-related outcome measure
mRS	Modified Rutgeerts’ score
HSEEA	Hand-Sewn End-to-End Anastomosis
SSSA	Stapled Side-to-Side Anastomosis
CDAI	Crohn’s Disease Activity Index
